# What the Body Reveals about Lay Knowledge of Psychological Flexibility

**DOI:** 10.3390/jcm11102848

**Published:** 2022-05-18

**Authors:** Neal Falletta-Cowden, Patrick Smith, Steven C. Hayes, Sandra Georgescu, Seyed Ali Kolahdouzan

**Affiliations:** 1 Department of Psychology, University of Nevada, Reno, NV 89557, USA; patricks@nevada.unr.edu (P.S.); hayes@unr.edu (S.C.H.); 2 Contextual CBT Center, Los Altos, CA 94022, USA; smgeorgescu@gmail.com; 3 Supportive & Palliative Care Department, Isfahan University of Medical Sciences, Isfahan 81746-73461, Iran; akolahdouzan@gmail.com

**Keywords:** psychological flexibility, embodied knowledge, bodily expression, physical metaphor

## Abstract

The embodied knowledge of psychological flexibility processes was tested by examining the ability of raters to score whole body pictures based on the degree to which they were open, aware, and engaged. Participants’ best and worst physical posture was photographed when asked to think of a difficult psychological matter. Naïve and untrained raters (*n* = 16) showed excellent reliability while rating the postures of 82 persons from the general population in Reno and Chicago in the USA and recent Iranian immigrants in the Maryland/DC area. Participants showed embodied knowledge of psychological flexibility concepts across all three locations (though significantly less among those recently from Iran). Thus, experience alone appears to teach people that psychological flexibility is helpful, even if they are unable to express this knowledge in words. Implications for psychotherapeutic work is considered.

## 1. Introduction

Psychological flexibility refers to a set of processes relevant to psychological health that are particularly targeted by Acceptance and Commitment Therapy (ACT) [1] and by several other intervention methods, especially those grouped under the label of “third wave” cognitive behavioral therapy [2]. Changes in psychological flexibility or its elements are consistent predictors of mental and behavioral health when examined in longitudinal correlational studies [3] and in country-wide representative samples [4]. A recent meta-analysis of mediational analyses [5] found that psychological flexibility and its elements were consistent mediators of ACT outcomes and, at times, also mediated other forms of behavioral and cognitive therapy (see also [6]). Across the entire mediational literature, psychological flexibility processes appear to be among the most common mediators of psychological interventions [7].

Psychological flexibility can be summarized as learning to be more emotionally and cognitively open; more consciously aware of the present moment, both internally and externally; and being more actively engaged in a values-based approach to living [1]. Hayes et al. [2] found that virtually all more recent forms of cognitive behavioral therapy (CBT) include some treatment elements targeting being more open, aware, and actively engaged in life. Despite that pervasive awareness, the culture at large at times seems surprised at the idea that positive outcomes could be achieved by being less avoidant of psychological pain and vulnerability, less focused on being right, or less attached to one’s self-conceptualization, to pick just three common forms of inflexibility. Given that targeting flexibility processes is helpful in a such a wide variety of areas of human functioning, however (see [8] for a recent book length summary; see [9] for a meta-analysis of recent meta-analyses of ACT), one wonders why this is.

In principle, life experience alone should be a more skillful teacher of flexibility processes. After all, people should have had scores, if not hundreds, of exemplars in their lifetime, positive and negative, of the relative value of flexibility over inflexibility. For example, most people should have had moments in their lives when they attended to difficult feelings instead of ignoring or suppressing them, took corresponding action even if difficult, and then experienced better long-term outcomes from this instance of relative emotional openness. When combined with multiple exemplars of the pervasive cost of suppression of difficult thoughts or avoidance of difficult feelings [10], it would seem that people should be wiser from experience.

One possibility is that most people *do* learn these life lessons, at least to some degree, but they cannot readily access this knowledge verbally. Knowledge shaped by experience is often hard to put into words. For example, it has long been established that people can respond physically to contingencies they are totally unable to verbalize [11]. Similarly, a person may be able to show others how to walk, swim, or dance by demonstration but be completely unable to describe or explain these actions in detail when asked to do so with words. In the case of psychological flexibility, recognizing and verbalizing such knowledge may also be actively discouraged by culturally common inflexibility messages, such as “don’t worry about it”, “stop thinking about it”, “don’t let them see you sweat”, and so on. If such issues are the difficulty, and not learning by experience per se, it is possible that people may be able to show knowledge of psychological flexibility using non-verbal means, even if they cannot do so descriptively.

The present study examined the possibility of widespread embodied knowledge of psychological flexibility. We are using the term “embodied” in the definitional sense: “To give a bodily form to; incarnate” [12]. People can use their body to express complex ideas through metaphors even when words fail [13,14,15,16] and can express complex dispositions with their body and head alone [17]. Research shows that “acted out” or embodied metaphors are also more likely to be perceived as apt, to be remembered, and to be used to guide behavior when compared to verbal instruction [18,19,20]. Their utility may help explain why clinical metaphors applied to psychological change [21] are often embodied.

In psychotherapy, embodied metaphors are often used to help bring psychological flexibility messages home. For example, a hand placed in front of one’s face may be used to demonstrate cognitive fusion or dropping a tug-of-war rope can express acceptance (e.g., [22]). Intentionally using metaphors that highlight bodily impact as a relational ground [23] has been empirically established as beneficial in psychotherapy (e.g., [18,20]).

The present study asked two distinct questions about embodied knowledge of psychological flexibility. First, we examined whether untrained individuals can reliably use psychological flexibility terms to evaluate the meaningful bodily positioning of others. Second, we asked whether people would metaphorically display psychological flexibility or inflexibility with their body when expressing best and worst mental postures toward the same psychologically challenging event. If the answers to both of these questions are “yes,” it suggests that, at least to some degree, people have learned the life lessons of psychological flexibility and can express and recognize these lessons in bodily form, despite the common inability to verbalize flexibility concepts as being critical to personal psychological health or to display such skills when needed in a challenging situation.

## 2. Materials and Methods

### 2.1. Participants and Procedure for Collection of Photos

The participants in the current study included a convenience sample of 98 total individuals: 82 photo participants and 16 photo raters. Photos of participants included 31 adults from Reno, Nevada; 32 individuals from Chicago, Illinois; and 19 recent immigrants from Iran (dominantly from Tehran) living in the Washington, DC and Maryland area. Participants were recruited through a mixture of word of mouth and online recruitment software. Differences in Institutional Review Boards and photo settings across the sites produced minor differences in procedures; all recruitment announcements stated that the purpose of the study was to investigate whether individuals’ body posture could reveal emotion, cognition, or other psychological experiences (for Iranian participants, those announcements were in Farsi). Of the photo participants, 28 were male, 53 were female, and 3 did not disclose their gender; 39% were university students. Photo participants from Reno, Nevada were compensated for their participation with class credit; participants from the other locations were entirely voluntary and did not receive any compensation for their participation. Since this is the first study of its kind, site selection was based on a common-sense desire to vary locations enough to see how universally psychological flexibility processes applied to the task (recent Iranian immigrants were used because travel restrictions to Iran made onsite work there impossible). There was no other theoretical basis for site variation.

Photo participants met with an experimental assistant, and after providing informed consent, they were taken to a setting in which a picture could be taken. The settings differed somewhat in the three locations. Reno participants came to the research team’s laboratory space and were photographed in a small (~160 sq ft) research office in front of a plain light-colored wall without access to any furniture. Chicago and Iranian research team members photographed participants at a location of the participant’s choosing, resulting in a wider variety of natural settings and furniture. Participants were then given the following written instructions (translated into Farsi for those participants from Iran):

“We are interested in seeing how people embody how they deal with psychological issues. Imagine that you are a great sculptor and that you can use your amazing sculpting skills to shape your body so that anyone looking at it would understand what it is like to be you when dealing with some psychologically challenging issue. We even have the phrase “what is your posture toward that issue,” so put your body in a position that would show the following:

Please think of something that is a major challenge for you at times inside the skin. It could be a difficult or self-critical thought, a painful feeling, a difficult memory, a sensation, or a behavioral urge, and physically put your body in a posture that shows you at your worst when dealing with this issue. You do not have to tell us what the issue is, but keep it in mind as you show us, with your body, you at your worst in dealing with this issue.”

A photo was then taken of the participant. The following instruction was then given:

*“Then, please think of a time when you are at your very best when dealing with this very issue. Again, keep the specific issue in mind as you show us you at your best in dealing with it; put your body in a posture that expresses that.”*

A second photo was then taken.

Photos were taken of the worst posture first, since that pose would be more consonant with painful or difficult experiences and, thus, might make the task easier to understand initially.

### 2.2. Participants and Procedure for Collection of Ratings

All photo raters were undergraduate or graduate research assistants recruited through word of mouth within the University setting at either University of Nevada, Reno (*n* = 14) (received academic credit for their work) or the Chicago School of Professional Psychology (*n* = 2); 3 were male, 11 were female, and 2 photo raters did not disclose their gender. Photo raters were naive regarding the purposes of the study and were not deliberately trained regarding psychological flexibility processes and terms in the context of this study.

Photo raters were provided with instructions and surveys via online software at either the Reno or Chicago location. Ratings were completed in single sessions with batches of photos presented one at a time. The batches were presented across two to four sessions per rater based on random assignment. Photo raters were given the following instructions:

Thank you for volunteering!

Below are four links to different image sets to be rated. Please start with the leftmost link and work your way right. Please do not distribute or discuss your links/images with anyone else. I will debrief you on the experiment once you have completed all the links.

Once you begin rating a set (each link is a set), there is no save and return. 

Please have at least an hour available for each of the first two links and 90 min for the each of the second two.

Every image has multiple sliders for rating. Each slider must be moved for the value to be recorded. That means that if you want a middle value (where the slider starts by default), you need to move the slider to either side and then back.

There are text input boxes in some of the sets. You do not have to write anything, but you can if you want.

The photos then appeared one at a time on screen. Via software, three continuous sliders were shown on screen to rate each photo in sequence. The slider began at the mid-point (0) on a −10 to 10 scale with unique labels for each slider: “closed” (−10) to “open” (10); “unaware” (−10) to “aware” (10); and “inactive/disengaged” (−10) to “active/engaged” (10). To ensure engagement with the rating program, photo raters were required to move each slider off of the 0 mark for their ratings to be recorded. As indicated in the instructions to raters, they were able to return the slider to the 0 mark if that was their active choice. No definitions or examples were provided of what these terms meant as applied to pictures; the labels themselves were the entirety of the guidance received.

“Best” and “worst” pictures were mixed together, and each rater scored all 164 whole body pictures in a random order unique to them. In other parts of the overall study not reported here, raters looked at only faces cropped from photos or only bodies without faces. Additionally, all raters saw the same pictures but with scales that prompted them to make specific ratings of body parts (e.g., head tilt, hands, etc). The order of these other rating experiences were controlled and counterbalanced with the ratings reported here. The results of those body part-specific analyses will be reported elsewhere.

### 2.3. Data Analytic Approach

We first assessed whether ratings of pictures using naïve untrained raters and simple verbal labels for the “pillars” of the psychological flexibility model (open, aware, and engaged) would lead to reliable ratings of whole-body photographs. The raw data examined in the study consisted of scores that ranged from −10 to 10 for closed to open, unaware to aware, and inactive/disengaged to active/engaged. The reliability of these three measures was examined by calculating intraclass correlation coefficients (ICCs) for each, across all 16 raters and all 164 whole-body photographs evaluated in the study. This question needed to be considered first, because assessing the impact of pose instructions required having a usable measurement system. We then used Chronbach’s alpha to assess whether these three measures themselves covaried in ways that suggested a common core concept, namely, embodied psychological flexibility.

The main question asked in the present study was whether a convenience sample of participants across three sites would display different levels of psychological flexibility or inflexibility using only their body when expressing best and worst mental postures to psychologically challenging events. This was examined using paired associate t-tests across the measures. In effect, this analysis treated pose instructions as an independent variable assessed within person. If people learn the relevance of psychological flexibility by experience, it would be predicted that the “best” photos would be more open, aware, and engaged.

Testing for differences between the three samples was not a main focus of the study, but it was tested using a repeated measures ANOVA that entered location as a between factor and pose instructions as the within factor across the various measures.

Data analyses were conducted using SPSS version 27, except as noted. Cutoffs for verbally characterizing effect sizes followed the conventions set by Cohen [24].

## 3. Results

An important question asked in the current study was whether untrained raters could reliably use terms for the pillars of psychological flexibility as rating guides. They were able to do so. Results found ICC values (applying a two-way mixed model for absolute agreement) of 0.963 for open (95% CI: 0.954–0.971), 0.959 for aware (95% CI: 0.948–0.968), and 0.951 for engaged (95% CI: 0.938–0.961). Applying widely accepted cutoffs for ICC values [25], even the lowest values within these three 95% confidence intervals would still indicate “excellent reliability” for each of the three ratings, a term that is generally applied to ICCs above 0.90. Given the high ICCs, in the rest of the study, the degree to which particular images were said to be open, aware, or engaged were based on mean ratings in these areas across the 16 raters.

Cronbach’s alpha was then examined across the three ratings of elements of psychological flexibility for the 164 whole-body photos to assess whether they reflected a common core concept. Cronbach’s alpha was 0.965, above the cutoff of 0.95 suggested even for highly refined scales [26]. This indicates that the three scores (open, aware, and engaged) could indeed be treated as a composite. These scores were thus calculated for each photo, based on a simple average of the three ratings. Like the three ratings themselves, composite scores could, in principle, vary from −10 to 10; in this study, the actual range across all whole-body photos was −8.92 to 8.15 (Mean = 0.24; SD = 5.06).

**Figure 1:**
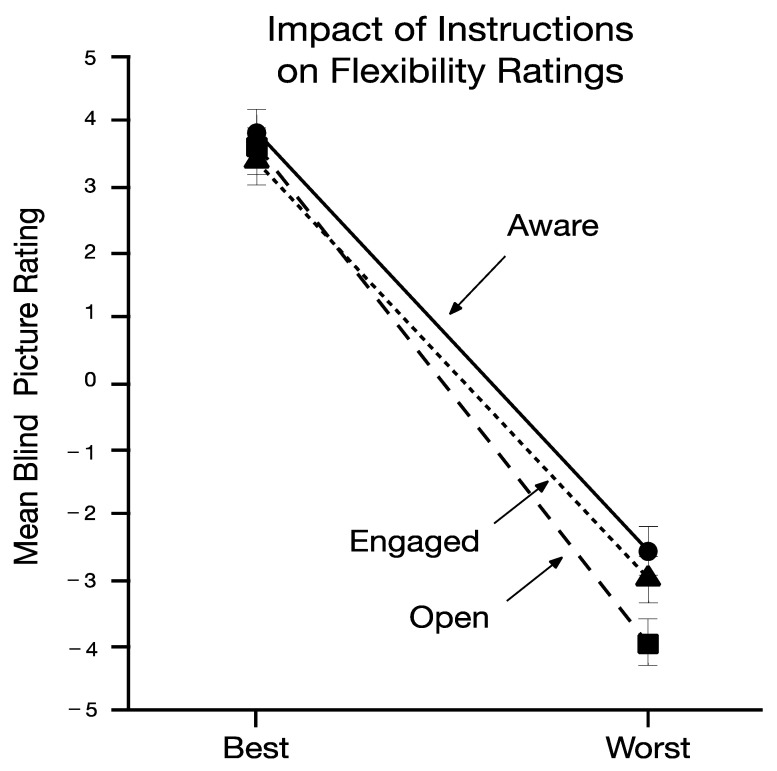
Mean open, aware, and engaged ratings for best and worst poses.

The main question asked in the present study is whether a convenient sample across three sites would display different levels of psychological flexibility or inflexibility using only their body when expressing best and worst mental postures to psychologically challenging events. Paired associate t-tests conducted on the composite scores for the “best” poses as compared to the “worst” poses showed a large and statistically significant difference (*t* (82) = 12.64, *p* < 0.0001, d = 1.40; Best mean and SE = 3.65 (0.40); Worst mean and SE = −3.82 (0.42)). When analyses were conducted of individual items’ ratings, pictures of the best pose as compared to the worst pose were seen to be more open (*t* (81) = 14.00, *p* < 0.0001, d = 1.55), aware (*t* (81) = 10.80, *p* < 0.0001, d = 0.91), and engaged (*t* (81) = 11.01, *p* < 0.0001, d = 0.93). These results are shown in Figure 1. When physically displaying their psychological postures toward difficult moments, naïve participants’ body posture metaphorically reflected the three pillars of psychological flexibility in the eyes of blind untrained raters.

**Figure 2:**
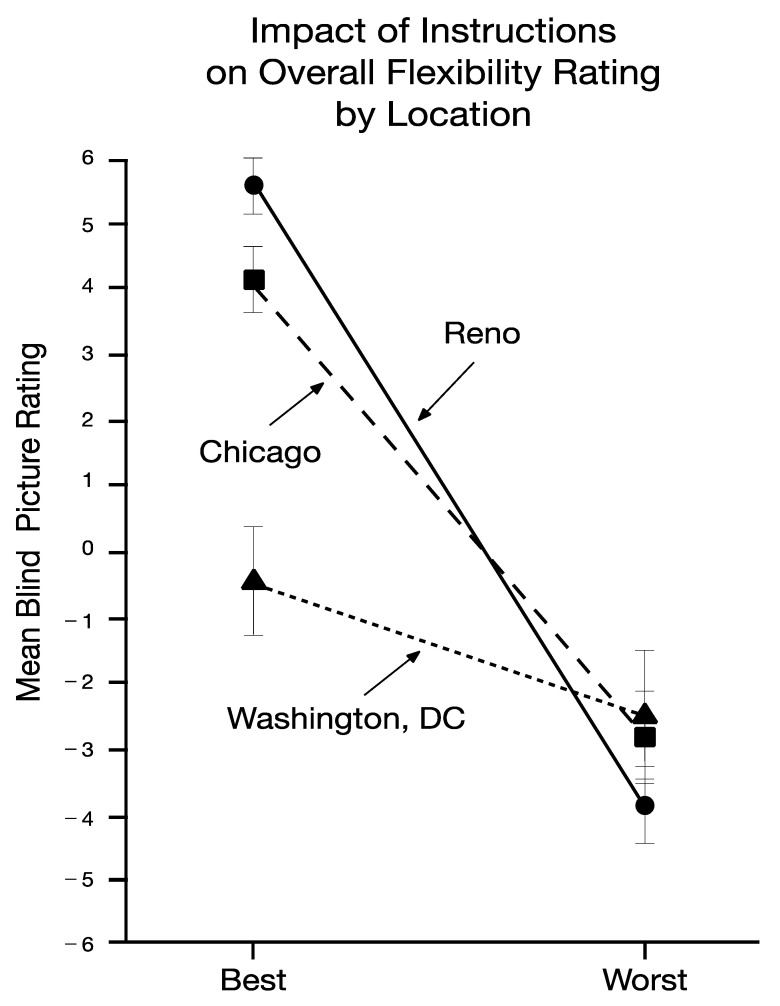
Mean composite scores for the best and worst poses in Reno, Chicago, and Washington, DC.

To see if these results applied across the three samples, a repeated measures analysis of variance was conducted on the composite scores, with location as a between-subject factor and pose as a within-subject repeated measure. Results showed a large and significant effect for pose ((*F* 1,79) = 184.19, *p* < 0.0001, partial eta sq = 0.70), a small and significant effect for location ((*F* 2,79) = 5.17, *p* = 0.008, partial eta sq = 0.12), and a medium and significant effect for their interaction ((*F* 2,79) = 20.73, *p* < 0.0001, partial eta sq = 0.34). The nature of the interaction can be seen in Figure 2. Post-hoc comparisons found significant (*p* > 0.05) pose effects on the composite scores at all three locations (Cohen’s *d* = 2.47, 1.59, and 0.57, for Reno, Chicago, and Washington D.C., respectively), but the “best” photos of Iranian immigrant living in Washington, DC differed from the other populations.

To explore this interaction further, univariate repeated measures ANOVAs were conducted for each of the three specific ratings, treating location as a between-participants variable and body pose as a within-person variable (see Table 1). For each measure, there was a significant and medium overall interaction between the location of the sample and pose instructions (open: *F* (2.9) = 19.35, *p* < 0.0001, partial eta sq = 0.33; aware: *F* (2.79) = 14.2, *p* < 0.0001, partial eta sq = 0.26; engaged: *F* (2.79) = 19.26, *p* < 0.0001, partial eta sq = 0.33). Post hoc comparisons of mean values for the “best” and “worst” poses showed very large (Cohen’s *d* > 1.20) and statistically significant effects across all three rating items in Reno and Chicago, but among Iranian immigrants, a medium and significant difference was found for “open” (*d* = 0.70, *p* = 0.007), a small and marginally significant effect was found for “aware” (*d* = 0.45, *p* = 0.066), and a small and non-significant effect was found for “engaged (*d* = 0.33, *p* = 0.17). Some of this difference appeared to be gender-based. Independent sample t-tests showed no gender difference in Reno or Chicago but found a large and significance difference among Washington, DC (i.e., Iranian immigrant) participants (*t* (16) = −2.42, *p* = 0.028, *d* = −1.14). Iranian females showed a difference in the expected direction in the composite score between the “best” and “worst” photos (Mean difference for “worst” pose = −4.02, SE = 1.19), but males did not (Mean difference for “worst” pose = −0.31, SE = 0.97). While the sample is too small for a more detailed analysis, some Iranian males closed their eyes and clasped their hands as if in prayer in the “best” photo, a physical posture that might lead to lower aware and engaged ratings.

Overall, 75 of the 82 photo participants (91.4%) showed lower composite scores in the worst pose versus the best pose. All seven who did not were male, and six of those seven were from Iran.

## 4. Discussion

Across most forms of so-called “third wave” CBT, psychological flexibility processes are often separated into greater openness, awareness, and active engagement [2]. Although people commonly claim that psychological flexibility concepts are counterintuitive, the present data suggest that most lay members of the public already have embodied knowledge of psychological flexibility. The evidence for this is twofold. First, the present research shows that the terms “open,” “aware,” and “active” can be used very reliably by naïve and untrained raters to rate the “body language” of others. Furthermore, people generally show physical indications of greater openness, awareness, and active engagement when asked to show themselves at their best and worse when dealing with psychological issues. This raises the interesting possibility that people have typically learned about psychological flexibility experientially, even if they are unable to express that knowledge verbally. The purpose of the current paper was to explore this possibility, and below, we will further illustrate the possible implications of individuals possessing this knowledge.

**Figure 3:**
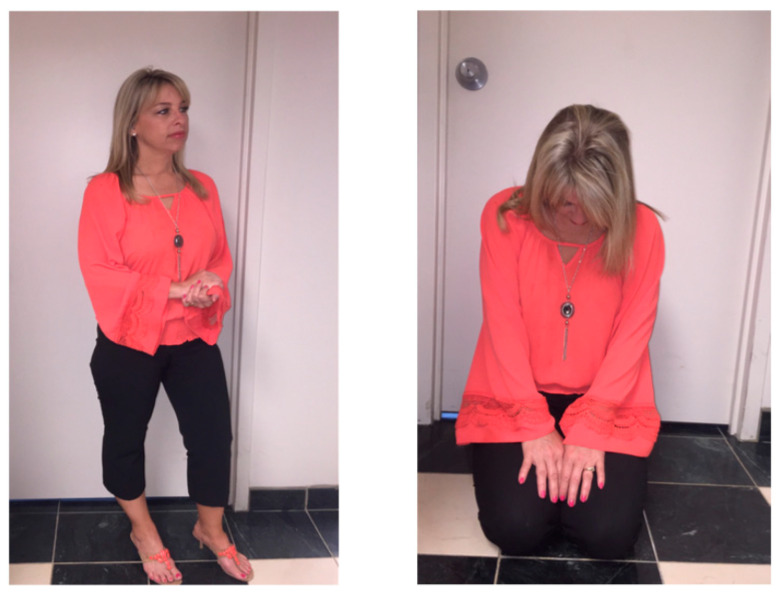
Example of a best (**left**) and worst (**right**) pose.

Figure 3 (shown with permission of the participant) is characteristic of the types of poses people assumed under the instructional conditions tested in the current study. In the “best” photo on the left this participant has her head up and her eyes open; her feet are apart; and her elbows are wide. In the “worst” photo on the right her head is lowered; her eyes are hidden; and her knees and elbows are pulled toward her body. Putting that posture into words, she is obviously less open, aware, and engaged with the world around her at her worst and is more open, aware, and engaged at her best.

It is not immediately clear how to interpret the differences between locations. Traditional self-reports of psychological flexibility appear to be robust across cultures (e.g., [10]), but that may not apply to embodied knowledge. Since Iranian immigrant males showed a lack of differences between poses, gender-linked cultural differences need further investigation. The newness of the U. S. environment could have led to a more observational or restricted stance. These differences could also be due to cross-cultural effects between participants and raters. The photo raters from the United States may have had a harder time “reading” photos from persons recently from Iran. Cultural experience is known to impact the understanding “bodily dialects” in intercultural non-vocal communication [27,28], and a wider range of raters should be used in the future.

There are many possible applied implications that are worth exploring. The present results lend support to embodied work, suggesting that psychotherapists should attend to the degree to which clients’ postures are open, aware, or engaged and use that information in much the same way as practitioners attend to word usage, paralinguistic cues, rate of speech, and other sources of information about psychological states that can be sensed in real time. For example, if a therapist sees that their client hunches their shoulders and looks downward when emotion material comes up, it may be time to explore the degree to which the person is closed off to their emotions. In a more process-based approach, the therapist may wish to ask the client to assume postures and notice their psychological impact or to use embodied knowledge as a form of communications, such as asking “can you show me with your body how you are dealing with this issue today?” or “can you express how you dealt with this issue over the last week using only your body?” If artfully deployed, deliberate use by therapists of their own body posture might help build a sense of shared understanding and alliance, adding embodiment to the range of variables relevant to the therapeutic alliance [29].

It seems possible that some therapeutic interventions or measures might be augmented to incorporate embodied psychological flexibility. For example, exposure therapy might be done while using a variety of bodily positions to help increase awareness and choice in the face of aversive experience. The generalization of outcomes to a variety of psychological postures might enhance their use post-intervention. The assessment of neurobiological responses while assuming different body postures may give useful clues regarding how to create a neurobiological state of safety during treatment, a variable of known relevance to clinical outcomes [30,31]. As has been done successfully in the assessment of pain [32], the selection of pictures of body postures might provide useful information with children or when language barriers are large (“can you point to which picture more accurately expresses how you are handing this issue?”).

This is the first study if its kind, and future studies will need to control the details of procedure somewhat more tightly. More controlled setting variables (e.g., taking all pictures against a blank wall) could be helpful in detecting cross-cultural differences, for example. A sample of convenience may not generalize to a representative sample. Since this is the first study of its kind, we were unable to conduct a power analysis to determine the best sample size, which is also a limitation. We did not vary the order of poses in taking the initial photos, and thus, the possibility of an order effect was not controlled.

Despite such weaknesses, the present study raises the provocative possibility that the essence of psychological flexibility is known to most people via incidental life experiences. That finding alone raises many possible avenues toward learning how to enhance these processes in peoples’ lives in a way that reflects their known utility.

## Figures and Tables

**Table 1:** Mean scores by measure, pose, and location.

Measure	Location	Pose	Mean	Std. Error
Composite	Reno	Best	5.68	0.374
		Worst	−3.86	0.618
	Chicago	Best	4.07	0.508
		Worst	−2.88	0.697
	Washington, DC	Best	−0.45	0.854
		Worst	−2.52	0.967
Open	Reno	Best	5.61	0.486
		Worst	−4.81	0.622
	Chicago	Best	4.18	0.493
		Worst	−3.54	0.632
	Washington, DC	Best	−0.41	0.63
		Worst	−3.41	0.807
Aware	Reno	Best	5.81	0.566
		Worst	−3.41	0.831
	Chicago	Best	4.33	0.575
		Worst	−2.01	0.844
	Washington, DC	Best	−0.34	0.734
		Worst	−2.3	1.078
Engaged	Reno	Best	5.64	0.564
		Worst	−3.38	0.694
	Chicago	Best	3.71	0.573
		Worst	−3.04	0.705
	Washington, DC	Best	−0.6	0.732
		Worst	−1.85	0.9

## Data Availability

The data presented in this study are available on request from the second author (PS). The data are not publicly available due to the set including full-body photographs of all participants that may be identified. Some participants consented to the research with explicit instruction that their images be distributed only to photo raters and research team members. Any photos in this manuscript were shared with the participant’s consent, and the second author can provide datasets cleaned of those images with restrictions on distribution.
